# Assessment of bone regeneration after maxillary radicular cyst enucleation with or without bone grafting materials: a retrospective cohort study

**DOI:** 10.1007/s00784-024-05612-7

**Published:** 2024-03-14

**Authors:** Giusy Rita Maria La Rosa, Carlotta Ylenia Priolo, Roula S Abiad, Virginia Rosy Romeo, Emanuele Ambu, Eugenio Pedullà

**Affiliations:** 1 Department of General Surgery and Medical-Surgical Specialties, University of Catania, Catania, Italy; 2 Endodontic Division, Faculty of Dentistry, Beirut Arab University, Beirut, Lebanon; 3 Unit of Endodontics and Restorative Dentistry, Department of Medical Biotechnologies, University of Siena, Siena, Italy

**Keywords:** Bio-oss, Retrospective cohort, Bone augmentation, Bone regeneration, Maxillary radicular cyst

## Abstract

**Objective:**

The limitations of spontaneous bone healing underscore the necessity for exploring alternative strategies to enhance bone regeneration in maxillary radicular cyst cases. This retrospective study aimed to assess the impact of a bone substitute material (i.e., Bio-Oss) on bone volume regeneration following maxillary radicular cyst enucleation using cone-beam computed tomography (CBCT).

**Materials and methods:**

Seventy-three patients with maxillary radicular cysts were divided into two groups: one undergoing guided bone regeneration (GBR) with Bio-Oss and absorbable collagen membrane (*n* = 35), and the other receiving cyst excision alone (*n* = 38). Volumetric measurements using Amira software on CBCT scans evaluated bone regeneration, with cystic lesion shrinkage rates calculated. Intergroup comparisons utilized independent sample t-tests (*P* < 0.05), and linear regression analysis assessed the influence of preoperative cyst volume and group on bone healing.

**Results:**

Both groups showed similar success rates in bone formation at the 12-month follow-up, with no significant differences between them (mean (SD), control: 75.16 (19.17) vs. GBR: 82 (20.22), *P* > 0.05). Linear regression analysis revealed a negative correlation between preoperative cyst volume and bone regeneration in both groups (*P* < 0.05).

**Conclusion:**

Bio-Oss may not significantly enhance bone augmentation in maxillary radicular cysts. In addition, preoperative cyst volume negatively affected the shrinkage rate of cystic lesions.

**Clinical relevance:**

Clinicians should consider patient-specific factors such as anatomy and lesion size when determining the need for bone substitute materials. Future research could focus on optimizing treatment protocols and alternative regenerative strategies to improve patient outcomes in maxillary cyst cases.

## Introduction

Odontogenic cysts, originating from the odontogenic epithelium, develop within the maxillary bones, typically in association with dental elements. The formation of these cysts is linked to changes in the proliferation or degeneration of this epithelium [1].

In the 2017 edition of the World Health Organization (WHO) classification of odontogenic lesions, radicular cysts were categorized within the group of inflammatory cysts [2]. Radicular cysts emerge as a consequence of chronic inflammation in the periapical granulation tissue, which is found in close proximity to the apex of teeth, whether they have undergone endodontic treatment or not, and when there is an infected root canal system [3, 4].

Radicular cysts are more commonly diagnosed in the maxilla, occurring nearly ten times more frequently than in the mandible [5, 6]. While most radicular cysts typically measure between 5 and 15 mm, those located in the maxilla can exceed 15 mm in size [7, 8].

Previous research has indicated that larger periapical lesions can be effectively managed through non-surgical endodontic approaches [9–11], particularly when there is direct communication with the root canal system, allowing for pus drainage through access cavity preparation (as in the case of apical granulomas or pocket cysts) [12]. However, true cysts, which are separated from the apical foramen and are entirely covered by an intact epithelium, may not respond well to non-surgical treatments [13, 14]. In such cases, the conventional approach involves the enucleation of bone cysts. Nevertheless, dealing with the residual bony cavity after enucleation presents a significant dilemma, particularly when deciding whether to use additional materials for defect filling [15].

Traditionally, clot formation has been the preferred method for cavity recovery [16]. However, questions have arisen regarding the effectiveness of spontaneous bone healing in the absence of bone-filling materials. Studies have reported varying rates of spontaneous bone healing, ranging from 25.85 to 76% after 6 months and from 43.46 to 81.03% after 12 months postoperatively in cases where no additional filling material was employed [16–18]. These diverse outcomes underscore the limitations of spontaneous bone healing, and therefore, alternative strategies to enhance bone regeneration should be explored [15].

One promising alternative involves the use of bone graft materials during surgery, a strategy that is gaining increasing attention. Autogenous bone [19], artificial bone substitutes [20], and a combination of them [21] are widely employed to enhance bone regeneration, offering potential solutions to address the limitations of spontaneous healing.

Bio-Oss (Bio-Oss®, Geistlich Pharma, Thiene, Italy) is a biomaterial composed of ossified collagen derived from bovine bone, renowned for its capacity to promote osteogenesis and neo-angiogenesis [15, 22, 23]. The osteoconductive properties and porous three-dimensional structure of Bio-Oss facilitate the adhesion and growth of bone cells, thereby stimulating the regeneration process. Additionally, its bioactive effects further promote osteogenesis and neo-angiogenesis, contributing to the formation of new bone tissue [22, 23].

To date, there are no studies that have investigated the influence of bovine bone derivatives on the bone regeneration of exclusively maxillary radicular cysts.

Thus, the objective of this retrospective cohort study was to assess the impact of bone substitute filling material (i.e., Bio-Oss) on bone volume regeneration following the enucleation of maxillary radicular cysts, utilizing cone-beam computed tomography (CBCT).

The null hypothesis would be that the use of bone substitutes for filling does not significantly affect bone volume augmentation.

## Materials and methods

This retrospective cohort study was planned and approved by the Ethics Committee of the University of Beirut Arab University, Beirut, Lebanon (2023-H-0136-D-R-0575).

### Records collection

This retrospective cohort analysis was conducted in one private dental clinic in Bologna, Italy using the software OrisDent (Orisline, S.r.l., Veneto, Italy). Records from January 2016 and May 2022 were reviewed using the keywords “oral surgery” and “radicular cysts” to identify all maxillary radicular cysts histologically diagnosed and surgically treated.

### Inclusion criteria

The following case selection criteria were used:

(1) Patients aged 18–75 years in good physical status (ASA I) and oral health; (2) a diagnosis of maxillary radicular cysts verified on CBCT without dimensions limitations; (3) focal teeth were preserved with root canal treatment (RCT); (4) CBCT scans at baseline and at 12 months post-intervention to monitor and verify the presence, location, and extent of the resorptive defects and the subsequent bone regeneration; (5) patients’ records presenting appropriate information on medical and dental history, surgical treatment performed, pharmacotherapy received and data from follow-up visit.

### Exclusion criteria

(1) Mandibular radicular cysts (2); patients receiving any drugs which may be alter bone metabolism (i.e., calcium, bisphosphonates, glucocorticoids, hormone replacement therapy) [15, 24–26]; (3) patients reported with uncontrolled periodontal and endodontic disease and other oral disorders; (4) patients who reported to smoke more 10 cigarettes/day (i.e., heavy smokers) [15, 27].

Among the 153 available cases for review, a total of 73 patients satisfied the inclusion criteria and were selected for the analysis. Of these, 35 underwent guided bone regeneration (GBR) simultaneously with cyst enucleation, while 38 underwent cystectomy without GBR. Demographic data including sex and age as well as medical information were extracted. In addition, dental history encompassing previous periodontal, orthodontic and restorative procedures were also taken into account.

The characteristics of radicular cysts surgically treated including the tooth location (i.e., anterior/posterior) as well as treatment recommended or provided, were also recorded.

The surgical procedure obtained informed consent from patients. Enucleation was conducted under local plexus anesthesia by the same surgeon. To minimize the risk of recurrence, cyst curettage was performed to ensure the complete removal of residual fragments, including apicectomy for focal teeth. Apicectomy consisted in resection and preparation of the last 3-mm of root using an ultrasonic tip (Satelec Endo Success - AS 3D) mounted on an ultrasonic handpiece and powered according to the manufacturer’s specifications and root-end sealing with Endosequance putty RRM [28]. After meticulous enucleation, the bony cavity was rinsed with a solution of dilute povidone-iodine and saline water (1:1) for 30 s. Specimens were preserved in formalin for subsequent pathological examination.

### Control group

Patients in the control group underwent surgical cyst excision receiving primary closure, without any additional procedure for bone regeneration.

### Guided bone regeneration (GBR) group

Guided bone regeneration (GBR) was carried out by using deproteinized bovine substitute (Bio-Oss®, Geistlich Pharma, Thiene, Italy) with a granulometry of 0.25–1 mm and an absorbable collagen membrane (OsteBiol Evolution, Tecnoss, Giaveno, Turin, Italy). In the GBR group, the bony cavity was filled with large particle Bio-Oss mixed with autologous blood, and covered with a custom-fitted 20 × 20 mm OsteBiol Evolution membrane to match the bone window shape. Adequate soft tissue decompression was performed to achieve tension-reduced suturing. In all cases, primary closure was achieved using 4–0 polytetrafluoroethylene (PTFE) sutures, which were removed between the 10th and 14th day postoperatively. Oral antibiotics (penicillin) were administered to all patients for at least 3 days postoperatively, with instructions to maintain oral hygiene and use 2% chlorhexidine gluconate mouth rinses three times daily for one week after the procedure. In addition, patients received the same drug therapy consisting of Cortisone 4 mg in the first two days after the operation. Subsequently, a gradual dose reduction was carried out over three days, for a total of five days of therapy. Ibuprofen was prescribed for the first two days after surgery (i.e., three pills every 8 h for the first two days and then reduced to two). Gastroprotection was also prescribed to all patients to minimize the gastro-harmful effects of the drugs taken.

### Volumetric measurement

**Fig. 1:**
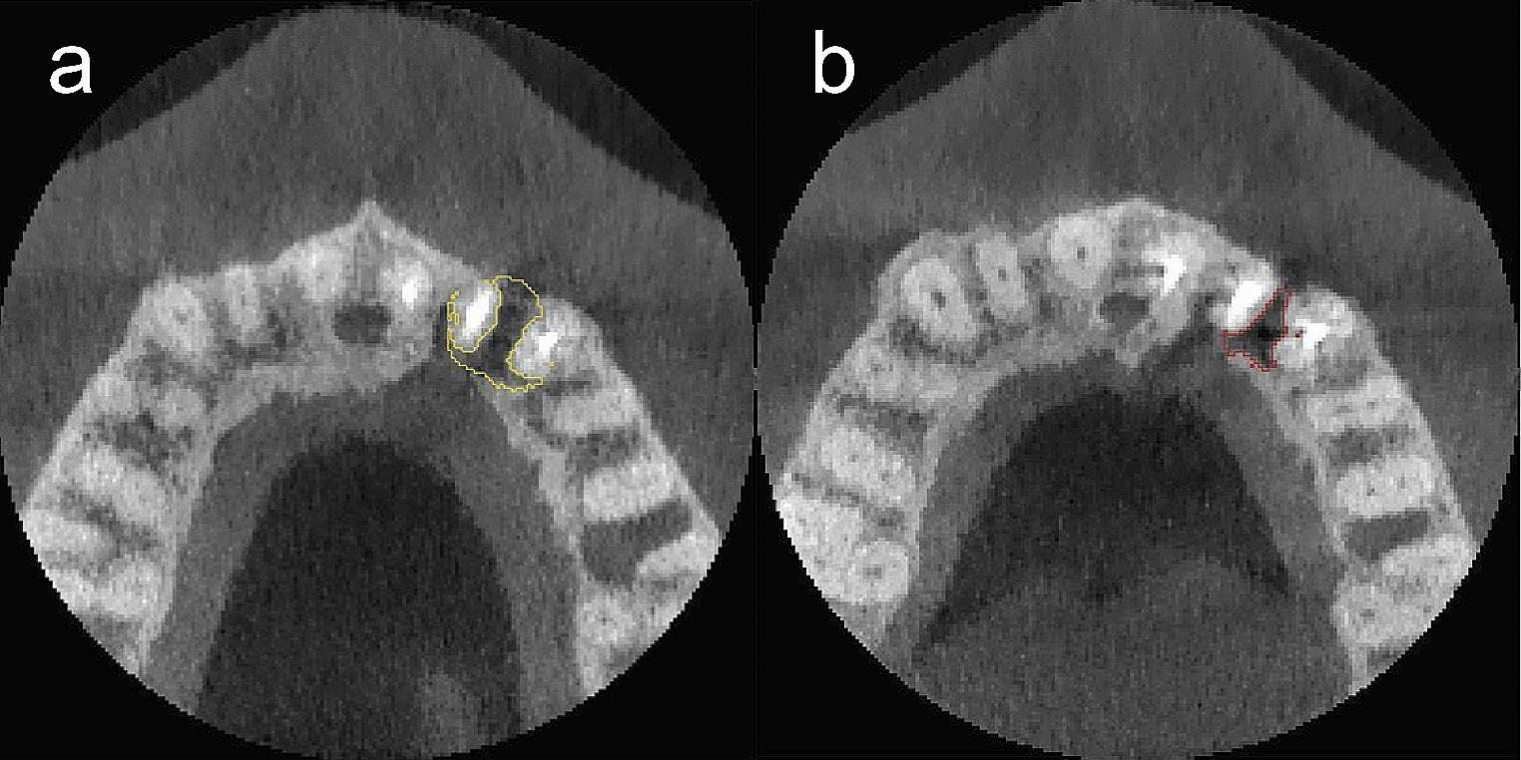
**a, b.** (**a**) CBCT capture of the area’s segmentation affected by the cystic lesion at baseline (in yellow) and (**b**) the bone cavity after 12 months post-surgery (in red) in control group

**Fig. 2:**
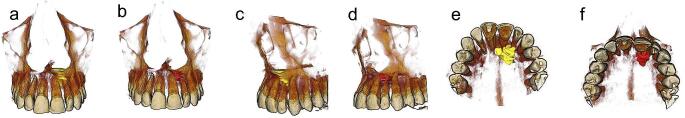
**a-f**. Three dimensional representations of cystic lesions at baseline (in yellow) and the residual bone cavity after 12 months post-surgery (in red) in GBR group. The visual representations were obtained using the Volume Rendering function with Surface View in Amira, allowing the view of lesion and bone cavity in various projections (a,b frontal; c,d sagittal; e,f axial)

Cone beam CT scans were acquired using KaVo OP 3D Pro (KaVo Dental GmbH, Germany) before the surgical procedure and 12 months postoperatively with a field of view varying from 5 × 5 cm to 13 × 15 cm diameter (scanning parameters: 90 kV, 5 mA, 8.14 s of exposure time, 0.38 mm voxel size). DICOM files from these two-time points were gathered and imported into Amira software (Thermo Fisher Scientific, Waltham, Massachusetts, USA). Using the Ortho slides and Surface area volume functions, the segmentation of the lesion was carried out, delimiting the area affected by the cyst (Fig. 1a) and cyst volume was automatically calculated (Fig. 2a,c,e). Following a methodology previously described [15], in the control group’s post-surgical images, the area of greatest radiolucency within the primary cavity was recognized as the residual bone defect. Its volume was assessed using the aforementioned method, setting a distinct boundary at the edge of the newly formed bone. Conversely, for patients who underwent GBR, the bone graft’s core displayed increased density or a heterogeneous density due to the graft particles. This core region was not included in the estimation of the postoperative reduction in space. The same procedure was performed on the 12-month follow-up CBCT image to quantify the extent of bone regeneration replacing the cystic defect (Figs. 1b and 2b, d and f).

The shrinkage rate (SR) of cystic lesions was calculated using the formula (preoperative cyst volume – 12 months postoperative defect volume) / preoperative cyst volume * 100% [15].

### Statistical analysis

The normality of data was tested by the Shapiro-Wilk test. Independent sample t-test test was applied for intergroup comparisons. Furthermore, preoperative cyst volume and group were included as predictor variables of shrinkage rate of cystic lesion in a linear regression model. The statistical analysis was performed using the software Stata version 17 (StatsCorp; TX; USA) with the significance level set at *P* < 0.05.

## Results

The dataset comprised records from 73 patients, with a total of 73 maxillary radicular cysts under consideration. Among these patients, 35 had undergone concurrent GBR alongside cyst enucleation (with ages ranging from 23 to 69 years). The remaining 38 patients, who had received cystectomy without GBR, constituted the control group (with ages ranging from 21 to 72 years).

With a sample size of 73 patients, a number of 2 groups, a power of 0.80, and α = 0.05, the present sample size was adequate to detect a minimum effect of f = 0.66 which corresponds to a medium effect according to Cohen’s recommendations. The analysis was performed with G*Power 3.1 for Macintosh (Heinrich Heine, Universität Düsseldorf, Dusseldorf, Germany).

A comprehensive overview of the patients’ general information, anatomical distribution, and pre-operative cyst volumes is displayed in the Table 1. The two groups were comparable for gender and age at baseline (*P* > 0.05). All patients experienced successful primary healing with minimal complications, primarily mild pain and swelling. No infection, rejection, adverse reactions, wound dehiscence, or material extrusion were recorded.

**Table 1:** Characteristics of the included patients

	Control Group (*n* = 38)	GBR Group (*n* = 35)
**Age (y, range)**	21–72	23–69
**Sex (n)**		
**Male**	21	16
**Female**	17	19
**Location (n)**		
**Anterior**	15	14
**Posterior**	23	21
**Pre-operative volume, mm** ^**3**^ **mean, (SD), [IQR]**	1355.36^a^ (288) [1288.21–1402.65]	987.59^a^ (201) [947.65–1167.54]

Same superscript lower letters indicate no statistically significantly difference (*P* > 0.05)

*IQR: interquartile range; n: number; SD: standard deviation*

The shrinkage rates at 12 months postoperatively are reported in the Table 2. Both the control group and the GBR group demonstrated stabilized bone formation at the 12-month follow-up, with no significant differences between them (*P* > 0.05).

**Table 2:** 12-month postoperative shrinkage rate (SR) (%)

	Control Group		GBR Group	
	Mean (SD)	Range	Mean (SD)	Range
**12-month SR (%)**	75.16^a^ (19.17)	57–91	82^a^ (20.22)	61–98

Same superscript lower letters indicate no statistically significantly difference (*P* > 0.05)

*GBR: Guided Bone Regeneration, SD: standard deviation*

As reported in Table 3, the linear regression analysis suggests that a statistically significant negative relationship occurred between the preoperative cyst volume and the shrinkage rate of cystic lesion (*P* < 0.05). The group variable was not a significant predictor of SR (*P* > 0.05).

**Table 3:** Linear regression model for preoperative cyst volume and group variable relevant to bone formation rate at 12 months

	Coefficient	SE	t	P>|t|	R^2^
**Shrinkage rate**					*0.270*
**Preoperative cyst volume**	*-0.017*	*-0.007*	*-2.32*	*0.030*	
**Group**	*5.345*	*6.898*	*0.77*	*0.447*	

*SE: standard error*

## Discussion

The current study investigated the effects of a bone substitute material on bone volume regeneration after maxillary radicular cyst enucleation using CBCT.

Our methodology involved the application of Bio-Oss and an absorbable membrane (i.e., Bio-Gide) following cyst enucleation. The use of regenerative materials after the surgical removal of maxillary cysts lead to several advantages, including stimulating bone regeneration, reducing the risk of surrounding bone resorption, and improving the overall healing process post-surgery [15, 21]. Furthermore, the application of such materials supports soft tissue regeneration and contributes to the anatomical reconstruction of the maxillary area, providing benefits in terms of both functionality and aesthetics for the patient [15]. Autologous bone grafting currently represents the best grafting material because of its high osteogenic, osteoconductive, and osteoinductive potential [29]. However, autologous bone extraction requires an invasive procedure, and sometimes a second surgical intervention is necessary to collect the donor tissue [30]. Alternatively, bovine bone has proven to be a valid therapeutic approach in bone tissue regeneration as evaluated through computer tomography [15, 22, 23]. The specific volume of Bio-Oss was tailored to match the cavity size, with membranes used to cover the bone window. The membranes used in the study group contributed to protecting and stabilizing the regeneration area, thus facilitating the healing and bone regeneration process [31]. The use of CBCT ensured the precision and reliability of the findings obtained. CBCT, with its three-dimensional imaging capabilities, provides a comprehensive view of the maxillary region, allowing for accurate assessment of cyst characteristics and bone regeneration [32, 33]. The detailed visualization facilitated precise measurements, contributing to a deeper understanding of the outcomes. CBCT, as opposed to traditional radiographic methods, offers enhanced diagnostic capabilities, ensuring a thorough evaluation of the treatment’s impact on bone density and structure [34]. However, CBCT measurements may overestimate buccal bone thickness and caution is required when using CBCT for measuring thin bone structures [35].

The current results showed that Bio-Oss and an absorbable membrane had no significant impact on bone regeneration after the enucleation of maxillary cysts. Thus, the null hypothesis cannot be rejected. These results differ from those of a previous study that reported significant benefits in jaw cysts treated with Bio-Oss [15]. This contrasting finding prompts a deeper examination of the specific dynamics of maxillary cysts. Potential factors contributing to this result may include the distinct anatomical characteristics of the maxilla, lesion sizes, and the follow-up duration. In particular, the study by Shi et al. [15] reveals notable differences. The baseline characteristics, including anatomical region and lesion size, play a crucial role in the varying outcomes observed. Particularly, both maxilla and jaw cysts, as well as larger preoperative cyst volumes, were included in Shi’s study [15]. Moreover, the SR was calculated at intermediate follow-ups (i.e., 3 and 6 months). Thus, it could be hypothesized that the advantage provided in bone healing by grafting materials may be more pronounced at short time intervals, while at 12 months, the healing is comparable between the two groups independently of grafting materials. More studies are needed to validate these hypotheses. The results of this study are consistent with some previous research that highlighted the possibility of achieving bone regeneration up to 99.72% at 12-month post-intervention without the use of biomaterials [36]. Moreover, a previous study reported complete bone healing in patients diagnosed with maxillary and mandibular cysts between 12 and 24 months after surgical intervention, without the use of biomaterials [17]. However, the patients were not treated with the same surgical technique, and the assessment of the residual cavity was performed using orthopantomography (OPG), which is less accurate for image evaluation [37]. Furthermore, the study included different histological cyst types, with only 5 of 44 patients diagnosed with radicular cysts [17].

Preoperative cyst volume was negatively correlated with SR of cystic lesion. In other words, as the volume of the cyst before surgery increased, the rate of shrinkage (and potentially bone regeneration) decreased. These findings are in agreement with previous results showing that cyst size had suppressive influence on bone formation [15]. Larger defects may lead to a prolonged remodeling process due to the increased time required for vascularization and bone formation, even when bone graft substitutes like deproteinized bovine bone are used as scaffolds to facilitate regeneration [15].

Acknowledging the limitations of our study is crucial. These include the retrospective nature, which cannot prove causality [38], a reduced sample size, and the absence of randomization. These factors introduce potential biases and limit the generalizability of the findings. Thus, further studies are necessary to confirm these results. The study did not evaluate the potential impact of gender or the anatomical position of the cyst (i.e., anterior versus posterior maxilla) on bone regeneration due to an inadequate sample size. Therefore, these factors remain unexplored, and further research with a larger cohort is warranted to determine their possible influence on the healing process after cyst enucleation. Additionally, the regeneration was evaluated at 12 months. Intermediate as well as longer follow-ups are necessary to evaluate potential differential regeneration speed between the two groups and representativeness of general population. Moreover, the results might differ in the case of other types of maxillary cysts, such as keratocystic odontogenic cysts or paradental cysts [26]. Finally, it is important to consider that volumetric measurements may be subject to a certain degree of error, influenced by the anatomical region, image quality, and variability in scan interpretation.

Despite these above-mentioned limitations, our study contributes valuable insights into the complexities of maxillary cyst treatment. It is important to emphasize that all selected patients had maxillary cysts of homogeneous histological nature (i.e., radicular cysts) and were treated by the same surgical team, using the same surgical technique and postoperative pharmacological therapy. This approach minimized possible distortions of results due to variations among patients.

The findings of this study have several notable clinical implications. Firstly, the observed lack of statistically significant difference in bone regeneration between maxillary cysts treated with and without substitute materials suggests that the universal application of Bio-Oss in diverse clinical contexts should be reconsidered. Clinicians should carefully assess the specific anatomical characteristics, lesion sizes, and follow-up durations of their maxillary cyst cases when determining the necessity for regenerative materials. Additionally, the study highlights the importance of tailoring treatment approaches to individual patient profiles, taking into account factors such as cost considerations and material availability, which can vary among regions and healthcare facilities.

Future research efforts could explore deeper into understanding the underlying mechanisms and factors influencing bone regeneration in the maxilla. Additionally, large prospective studies across various xenograft materials and alternative regenerative techniques such as stem cells [39] specific to maxillary lesions could provide valuable indications for optimizing treatment protocols and improving patient outcomes in these cases.

## Conclusions

In conclusion, no statistically significant difference emerged in bone augmentation of maxillary radicular cysts treated with Bio-Oss and absorbable membrane compared with those without substitute material. Furthermore, preoperative cyst volume negatively affected the bone healing. The lack of significant improvement in bone regeneration prompts a re-evaluation of the universal applicability of Bio-Oss in diverse anatomical regions and size lesions, emphasizing the importance of tailoring treatment approaches to specific clinical contexts.

## Declarations

### Data access statement

Research data supporting this publication are available from the corresponding author upon request.
